# Salivary and serum IL-10, TNF-α, TGF-β, VEGF levels in oropharyngeal squamous cell carcinoma and correlation with HPV and EBV infections

**DOI:** 10.1186/s13027-016-0093-6

**Published:** 2016-08-20

**Authors:** Małgorzata Polz-Dacewicz, Małgorzata Strycharz-Dudziak, Jakub Dworzański, Agnieszka Stec, Joanna Kocot

**Affiliations:** 1 Department of Virology, Medical University of Lublin, Lublin, Poland; 2 Department and Chair of Conservative Dentistry with Endodontics, Medical University of Lublin, Lublin, Poland; 3 Chair and Department of Medical Chemistry, Medical University of Lublin, Lublin, Poland

**Keywords:** Cytokines, Inflammation, Oropharyngeal cancer, HPV, EBV

## Abstract

**Background:**

Each year approximately 6,000 new cases of head and neck cancer are registered in Poland. Human papillomavirus (HPV) and Epstein-Barr virus (EBV) have been associated with tumour formation. Cytokines have been shown to play an important role both in inflammation and carcinogenesis and they can be detected in saliva and serum with ELISA assays. Salivary biomarkers may be used as markers of early cancer detection.

The aim of this study was the analysis of the serum and salivary levels of IL-10, TNF-α, TGF-β and VEGF in patients with oropharyngeal cancer and in healthy individuals. The level of these biomarkers was also analyzed in HPV- and EBV-related cases.

**Methods:**

The study involved 78 patients with histopathologically confirmed oropharyngeal squamous cell carcinoma and 40 healthy controls. Serum and salivary levels of IL-10, TNF-α, TGF-β and VEGF were analyzed both in patients and in healthy individuals by ELISA method using Diaclone SAS commercially available kits (France). EBV DNA was detected by the nested PCR for amplification of EBNA-2. HPV detection and genotyping was performed using the INNO-LiPA HPV Genotyping Extraassay (Innogenetics N. V, Gent, Belgium). The obtained results were subjected to statistical analysis using Mann–Whitney and Kruskal Wallis tests. Test values of *p* < 0.05 were considered statistically significant.

**Results:**

The level of tested cytokines was higher in patients than in controls both in serum (IL-10: 2.3 pg/ml vs 1.65 pg/ml, *p* = 0.0003; TGF-β: 11.3 ng/ml vs 7.8 ng/ml, *p* = 0.0005; VEGF: 614 pg/ml vs 210 pg/ml, *p* = 0.0004; TNF-α: 15.0 ng/ml vs 12.90 ng/ml, *p* = 0.1397) as well as in saliva (IL-10: 5.9 pg/ml vs 2.5 pg/ml, *p* = 0.00002; TGF-β: 24.1 ng/ml vs 14.8 ng/ml, *p* = 0.00002; VEGF: 4321 pg/ml vs 280 pg/ml, *p* = 0.0000; TNF-α: 23.1 ng/ml vs 11.3 ng/ml, *p* = 0.00002).

EBV DNA was detected in 51.3 % of patients and 20 % of controls, HPV DNA was present in 30.8 % of patients and 2,5 % of controls.

The level of IL-10 was statistically higher in patients infected with EBV, HPV and co-infected with EBV/HPV. The level of TNF-α was significantly higher in patients infected with EBV, while TGF-β in patients with HPV infection and EBV/HPV co-infection.

**Conclusion:**

Detection of salivary cytokines may be very helpful in early diagnosis, treatment and prognosis of OSCC.

## Background

Oral Squamous Cell Carcinoma (OSCC) as the most frequently diagnosed cancer in the head and neck region is a very important world-wide health problem [1]. About 90 % of all malignant tumours localised in the oral cavity and oropharynx are squamous cell carcinomas (SCC). Mortality for oral cancer is high because the early phase of the disease is often asymptomatic and most patients seek care with the late-stage symptoms [2]. Tobacco and alcohol are well-established risk factors for OSCC. Oncogenic viruses have also been involved in OSCC development [1, 3–8]. The International Agency for Research on Cancer (IARC) concluded that there is an evidence for a role of human papillomavirus (HPV), in particular HPV16, in the oropharyngeal cancer [9, 10]. A number of studies also suggest the role of Epstein-Barr virus (EBV) in the development of cancer in this region [11–14]. Many studies indicate that genetic mutations caused by oncogenic viruses lead to a different rates of tumour disease development. This effect is due to the varying levels of cytokines and other growth factors. These disorders lead to a faster (or slower) tumour growth as well as metastasis formation [15].

It has been shown that inflammation plays a key role in different stages of carcinogenesis. Cytokines are a group of host immune products involved in inflammation, immunity and defence against HPV infection, and modulation of HPV clearance plays an important role in oncogenesis [16]. Of particular interest has been the use of the salivary cytokine concentration as a marker of cell proliferation. The most studied cytokines include interleukin 10 (IL-10), tumour necrosis factor-alpha (TNF-α), vascular endothelial growth factor (VEGF) and tumour growth factor-beta (TGF-β). These salivary biomarkers were demonstrated to be increased in patients with oral cancer and several of them are still candidates for future clinical use [16–18]. Saliva is a body fluid very useful for the early diagnosis, monitoring and treatment of diseases [18]. Therefore, the aim of this study was to analyze the serum and salivary levels of IL-10, TNF-α, TGF-β and VEGF in patients with oropharyngeal cancer compared to healthy individuals. We also analyzed the level of these biomarkers in HPV- and EBV-related cases.

This research was approved by the Ethics Committee and is in accordance with the GCP regulations (no. KE-0254/133/2013).

## Methods

### Patients

The study was conducted among 78 patients with diagnosed and histopathologically confirmed OSCC hospitalized at the Otolaryngology Division of the Hospital in Radom, Poland. The patients had neither previous radiotherapy nor chemotherapy. The results were compared to 40 healthy controls. The control group involved persons hospitalized at the Otolaryngology Division due to diseases other than cancer. There were no statistically significant differences between the patients and the control group (age, sex, tobacco and alcohol consumption) (Table 1). Male patients dominated in the examined group (91.03 %). The patients’ ages ranged from 40 to 79 years. Most of the patients were aged 50–69 years (43.6 %), 15.4 % were under 50 years of age. Most studied individuals (64.1 %) lived in urban areas. A total of 79.5 % of the studied patients reported smoking, while 43.6 % pointed to alcohol abuse.

**Table 1:** Demographic and virological characteristics of patients and controls

		Patients		Controls		*P* value
		*N*	%	*N*	%	
Sex	female	7	8.97	5	7.5	>0.05
	male	71	91.03	35	92.5	
Age	40–49	12	15.4	7	17.5	>0.05
	50–69	34	43.6	19	47.5	
	70 +	32	41.0	14	35.0	
Place of residence	urban	50	64.1	26	65.0	>0.05
	rural	28	35.9	14	35.0	
Smoking	yes	62	79.5	32	80.0	>0.05
	no	16	20.5	8	20.0	
Alcohol	yes	34	43.6	20	50.0	>0.05
	no	44	56.4	20	50.0	
EBV DNA	positive	40	51.3	8	20.0	<0.001*
	negative	37	47.4	32	80.0	
HPV DNA	positive	24	30.8	1	2.5	<0.001*
	negative	54	69.2	39	97.5	

*statistically significant

### DNA extraction from fresh frozen tumour tissue

Fragments of fresh frozen tumour tissue (20 mg) both from the patients with OSCC and from the control subjects were cut and homogenized in a manual homogenizer Omni TH/Omni International/ Kennesewa/ Georgia /USA. DNA was extracted using a protocol as described in the DNeasy Tissue Kit Handbook (Qiagen GmBH, Hilden, Germany). Purified DNA was quantified by spectrophotometer (Epoch Microplate Spectrophotometer, BioTek Instruments Inc., Vinooski, Vermont, USA). The isolates were kept at −20 °C until the test was conducted. To verify the quality of the obtained DNA (presence of inhibitors of Polymerase Chain Reaction), assay of β-globin was performed.

### Serum collection

Venous blood samples from patients and control group were centrifuged at 1500 rpm for 15 min at room temperature and the serum was collected and frozen at −80 °C until analysis.

### Saliva collection

About 5 ml of unstimulated whole saliva was collected. The saliva samples were centrifuged at 1500 rpm at room temperature for 10 min and diluted (1:1) in PBS and frozen at -80 °C until analysis.

### Detection of viruses

EBV DNA detection: All polymerase chain reaction (PCR) reactions were carried out in the final volume of 25 μl using HotStartTaq DNA Polymerase (Qiagen, Germany). Concentrations of PCR reaction components were prepared as follows: 2.0 mM MgCl_2_, 0.2 mM dNTPs, 0.5 μM each forward and reverse primers and 0.5 U of HotStart Taq polymerase. During each run the samples were tested together with one negative (nuclease-free water) and one positive control (EBV-positive cell line, Namalwa, ATCC-CRL-1432).

Amplification of EBNA-2 gene: The nested PCR was carried out for amplification of Epstein-Barr nuclear antigen 2 (EBNA-2). The sequence of primers used for PCR was as follows: outer pair 5’ – TTT CAC CAA TAC ATG ACC C – 3’, 5’ – TGG CAA AGT GCT GAG AGC AA – 3’ and inner pair 5’ – CAA TAC ATG AAC CRG AGT CC – 3’, 5’ – AAG TGC TGA GAG CAA GGC MC – 3’. 2 μl of extracted DNA was subjected to the PCR mixture with the concentration as described above. The first-round amplification consisted of activation of polymerase 95 °C for 15 min, 35 cycles of 94 °C for 1 min, 55 °C for 1 min, 72 °C for 2 min and the final extension at 72 °C for 5 min. The second-round amplification was performed with 1 μl of first round PCR product in 30 cycles with an annealing temperature at 60 °C. The amplicons 368 bp, 473 bp in length (depending on the EBV type EBV-1 and EBV-2, respectively) were separated on 2 % agarose gel and purified using Gel-Out kit (A&A Biotechnology, Poland) for further analysis. Purified PCR products were sent to Genomed Warsaw company for sequencing.

HPV DNA detection: HPV detection and genotyping was performed using the INNO-LiPA HPV Genotyping Extraassay (Innogenetics N. V, Gent, Belgium; no cat. 81063) according to the manufacturer’s protocol. The kit is based on the amplification of a 65 bp fragment from the L1 region of the HPV genome. PCR products are subsequently typed with the reverse hybridization assay. The assay covers all currently known high-risk HPV genotypes and probable high-risk HPV genotypes (16, 18, 26, 31, 33, 35, 39, 45, 51, 52, 53, 56, 58, 59, 66, 68, 73, 82) as well as a number of low-risk HPV genotypes (6, 11, 40, 43, 44, 54, 70) and some additional types (69, 71, 74).

### Measuring of cytokines level

The levels of IL-10, TNF-α, TGF-β and VEGF were established in the saliva and sera of patients and healthy subjects by ELISA (enzyme-linked immunosorbent assay) using commercially available kits Diaclone SAS, France. The cytokine level of each serum sample was tested three times and the results are the means of these. The level of tested cytokines are expressed in pg/ml or ng/ml. The minimum detectable dose of IL-10 is less than 0.98 pg/ml (IL-10 HS ELISA kit) cat. no. 850.880.096; TNF-α – less than 8 pg/ml cat. no 950.090.096; TGF-β – 8.6 pg/ml cat. no. 650.010.096 and VEGF – 7.9 pg/ml cat. no. 650.080.096.

### Statistical analysis

To examine differences between the patients and controls in the distributions of demographic variables, smoking and drinking status, HPV and EBV status Pearson’s chi-square test was used. The Mann-Whitney test was used to compare the concentration of cytokines in patients and controls. The Kruksal-Wallis test was used to determine the association of the concentration of studied cytokines with such variables as smoking, alcohol abuse, histological grade (G) of the tumour, tumour (T) and node (N) stage according to TNM (tumour, node, metastasis) classification and presence of viruses. The observed differences were considered statistically significant at *p* <0.05.

## Results

The prevalence of HPV and EBV was higher in patients than in controls (*p* < 0.001). HPV DNA was detected in 30.8 % of patients, EBV DNA in 51.3 %, while in control group in 2.5 % and 20.0 %, respectively. Epidemiological, clinical and virological characteristics of patients are shown in Table 2. The level of all tested cytokines and growth factors was higher in patients than in controls both in serum and in saliva. The concentration of these biomarkers in the patients group was higher in saliva than in serum (Table 3). The level of IL-10 was statistically higher in smoking patients, in patients infected with EBV, HPV as well as in EBV-HPV co-infection (Table 4).

**Table 2:** Epidemiological, clinical and virological characteristics of patients (%)

		EBV		*P* value	HPV		*P* value
		Positive *N* = 40	Negative *N* = 38		Positive *N* = 24	Negative *N* = 54	
Sex	female	2.5	15.8	0.0401*	4.2	11.1	0.2908
	male	97.5	84.2		95.8	88.9	
Age	40–49	20.0	10.5	0.4902	0	22.2	0.5692
	50–69	40.0	47.4		75.0	29.6	
	70 +	40.0	42.1		25.0	48.2	
Place of residence	urban	70.0	57.9	0.2652	75.0	59.3	0.1736
	rural	30.0	42.1		25.0	40.7	
Smoking	yes	80.0	78.9	0.9083	83.3	77.8	0.5692
	no	20.0	21.1		16.7	22.2	
Alcohol	yes	45.0	42.1	0.7966	33.3	48.2	0.2196
	no	55.0	57.9		66.7	57.8	
Histological grading G	1	20.0	31.6	0.4911	33.3	22.8	0.0023*
	2	75.0	63.2		55.0	77.8	
	3	5.0	5.2		16.7	0	
T stage	1	10.0	15.8	0.0908	0	18.5	0.0004*
	2	45.0	42.1		75.0	29.7	
	3	30.0	10.5		8.3	25.9	
	4	15.0	31.6		16.7	25.9	
N stage	N0	50.0	63.2	0.7043	58.3	55.6	0.7948
	N1	20.0	15.8		16.7	18.5	
	N2	15.0	10.5		16.7	11.1	
	N3	15.0	10.5		8.3	14.8	

*statistically significant

**Table 3:** Serum and salivary level of cytokines in patients and controls

	Patients	Controls	*P* value
Serum			
IL-10 (pg/ml)	2.3 (1.2–3.9)	1.65 (0.54–2.9)	0.0003^a^
TGF-β (ng/ml)	11.3 (3.5–164.1)	7.8 (1.8–26.2)	0.0005^a^
VEGF (pg/ml	614 (14–1374)	210 (17–712)	0.0004^a^
TNF-α (ng/ml)	15.9 (4.9–39.1)	12.9 (10.4–19.5)	0.1397
Saliva			
IL-10 (pg/ml)	5.9 (2.9–12.7)	2.5 (0.45–5.1)	0.00002^a^
TGF-β (ng/ml)	24.1 (7–87.9)	14.8 (5.4–30.1)	0.00002^a^
VEGF (pg/ml)	4321 (1566–7791)	280 (130–980)	0.0000^a^
TNF-α (ng/ml)	23.1 (15.8–26.0)	11.3 (8.2–17.7)	0.00002^a^

median, test U Manna Whitney’a

^a^statistically significant

**Table 4:** Association of cytokines and growth factors levels with clinicopathological features

	IL-10	TNF-α	VEGF	TGF-β
Smoking	0.0017^*^	0.1542	0.4652	0.9853
Alcohol	0.1325	0.0915	0.1433	0.3278
Histological grade G	0.1105	0.0344^*^	0.0421^*^	0.7569
T stage	0.8636	0.0412^*^	0.0005^*^	0.2400
N stage	0.2592	0.3230	0.2878	0.0062^*^
EBV	0.0002^*^	0.0456^*^	0.0502	0.2842
HPV	0.0382^*^	0.7348	0.9016	0.0396^*^
HPV + EBV	0.0331^*^	0.6416	0.1180	0.0117^*^

^*^Statistically significant

There was a relationship between the concentration of both TNF-α and VEGF and the histological grade of the tumour (G) and the size of tumour (T stage), while TGF-β level was related to N stage. The level of the TNF-α was higher in patients infected with EBV, whereas TGF-β was higher in HPV and HPV/EBV co-infected patients. IL-10 concentration was higher in HPV, EBV as well as in HPV/EBV co-infected patients.

## Discussion

Various cytokines and growth factors play a significant role both in inflammation and carcinogenesis. Some cytokines are considered as pro-inflammatory (TNF-α, IFN-ɣ), whereas other are associated with anti-inflammatory effects (TGF-β) [2]. To the best of our knowledge, IL-10, TGF-β1, TNF-α and VEGF levels in patients infected with HPV and/or EBV have not been studied in the Polish population with oropharyngeal cancer.

Interleukin-10 (cytokine synthesis inhibitor factor, CSIF) is an important cytokine produced by several cells such as normal and neoplastic B cells, macrophages, T cells and some cancer cells [19, 20]. The immunosuppressive effects of IL-10 in the tumour environment have been repeatedly confirmed [21]. Our study demonstrated higher levels of IL-10 in saliva and serum samples of patients with OSCC than in control subjects. Moreover, IL-10 concentration in saliva was higher than in serum. Goncalves et al. [22] detected high expression of IL-10 in the tumour samples and elevated levels of this cytokine in saliva of patients with OSCC, which enabled to distinguish patients with cancer from healthy individuals. Jiang et al. [23] additionally studied correlation between IL-10 and advancing cancer lesions on animal models. In their research higher concentrations of IL-10 were associated with more severe disease and poorer prognosis of cancer. Lathers [24] observed an increase in the level of IL-10 with the formation of metastases in the lymph nodes. The influence of IL-10 on more severe cancer disease is explained by antagonistic effect of IL-10 on the formation of pro-inflammatory cytokines (IL-6, TNF, IL-1α, IL-1β, IL-12) and inhibition of the inflammatory response, which plays an important role in the development of cancer.

Tumour necrosis factor α (TNF-α) is a cytokine involved in systemic inflammation and is one of the cytokines that make up the acute phase reaction. TNF-α is secreted by macrophages, monocytes, neutrophils, T-cells, NK-cells following their stimulation by bacterial lipopolysaccharides [25]. It has a wide spectrum of biological activities including antitumour and antiviral activity. TNF-α may be involved in carcinogenesis through induction of proliferation, invasion, and metastasis because it may have both tumour-necrotic and tumour-promoting activities [26]. In our study a significantly higher level of this cytokine was obtained in the patients’ saliva comparing with the control group. In subjects with OSCC, TNF-α levels were higher in saliva than in serum. It may indicate the activity of the protein in close proximity to the tumour. Krishnan et al. [27] obtained significantly higher levels of TNF-α both in saliva and in the blood of the patients with OSCC. In a study carried out by Juretic et al. [28] salivary concentration of TNF-α was higher both in patients with premalignant oral lesions and in patients with OSCC as compared with healthy individuals. Rhodus [29], on the basis of his research analyzing the concentration of TNF-α in saliva, suggests that this cytokine can be used as a prognostic parameter for cancer.

TGF-β is a cytokine involved in various biological processes. The TGF-β pathway regulates many of the cellular processes and alterations in the signalling cascade may promote tumour development. The anti-proliferative activity of TGF-β1 is essential for maintaining normal tissue homeostasis and this activity of TGF-β1 is lost in many types of tumours [30, 31]. Some researchers point to an increase of TGF-β concentration in the case of tumour growth and they emphasize the prognostic features of this protein [32]. Other studies show no difference between the concentration of TGF-β in tissue samples and saliva [23]. These differences may come from the multiple effects of this cytokine on immune cells, the formation of blood vessels and fluctuation depending on the stage of cancer. Our study demonstrated significantly higher levels of TGF-β both in saliva and in serum of patients with OSCC. Moreover, salivary level was higher than serum concentration of this cytokine in the group of patients.

Because angiogenesis is essential for the growth and metastasis of tumours, we also investigated serum and salivary levels of VEGF (Vascular Endothelial Growth Factor), which is the main regulator of this process. In vitro experiments showed that VEGF stimulates mitosis of endothelial cells and their migration. VEGF may be also secreted by tumour cells and it acts in an autocrine manner to promote cell growth [33, 34]. In our study VEGF levels were significantly higher in patients with OSCC than in healthy controls both in serum and saliva. Similar results were obtained by other researchers [34–36], but there are also studies which demonstrated elevated salivary and serum levels of VEGF in OSCC patients without finding significant differences [37, 38]. Congruent outcomes were obtained by researchers who assessed the level of VEGF in tissue samples [39]. In a study carried out by Upile et al. [34] a direct correlation between T stage and N stage of the patients with OSCC and VEGF levels were found. Our research revealed a significant correlation between VEGF salivary and serum levels and clinicopathological features such as histology grading and T stage. These relationships are so important that some researchers suggest the possibility of using this protein as a tumour marker [35, 36].

As it is well documented, the main risk factors for head and neck cancer are tobacco, alcohol abuse and betel quid chewing but some types of head and neck SCC are linked to viral infections such as HPV and EBV.

HPV infection has been recognized as a necessary but not the only one cause of certain types of human cancers because cancer eventually develops only in a small group of individuals infected with HPV. Other factors are, therefore, necessary for malignant transformation of HPV-infected cells [21, 40]. Chronic inflammation by HPV with an inadequate host immune response may provide a potential for tumor formation. According to Chuang et al. [41], the development of HPV(+) OSCC is associated with IL-10 expression. These researchers demonstrated that HPV(+) patients with OSCC had higher IL-10 mRNA levels than HPV(−) patients. They also suggested that IL-10 may suppress T-cell immunity, which results in a persistent HPV infection. The correlation between higher levels of IL-10 in the group of patients with OSCC and the presence of HPV was also observed in our study. Moreover, the increase in the expression of IL-10 gene in people infected with HPV was demonstrated in the case of other types of tumours, such as cervical cancer [42].

VEGF is also a cytokine investigated toward its prognostic value in patients infected with HPV. Jo et al. [43] observed a significant increase in VEGF mRNA levels in HPV(+) tumours. Other researchers [44], however, did not obtain similar results. The present study also did not reveal such a correlation between VEGF and HPV status.

Many researchers found that HPV(+) and HPV(−) OSCC are two distinct forms of the disease. In general, HPV(+) cancer patients are younger, have a much better prognosis than HPV(−) [39, 41, 43, 45]. According to Chen et al. [45], high expression of IL-10 predicts a poor prognosis in OSCC. Chuang et al. [41] based on their research indicated that IL-10 mRNA expression levels may independently predict the survival and recurrence rates in patients with HPV(+) OSCC, but not in those with HPV(−) cancer.

Shi et al. [46] suggest that two DNA viruses, Epstein-Barr virus (EBV) and human papillomavirus (HPV), are associated with 38 % of all virus-associated cancers and the probability of one patient infected with two distinct types of viruses is increasing. During primary infection EBV infects oral epithelial cells and B lymphocytes. Like other herpesviruses, EBV has a productive lytic cycle and a latent form. Latently infected B cells can occasionally be stimulated to reactivate EBV [47]. Activation of the lytic phase or reactivation from latency is a key to virus transmission. Most diseases related to EBV infection are associated with the latent form of infection. Some of early products that further act as transactivators of the viral lytic phase are BZLF1 and BRLF1. BZLF1 plays important role in attenuating the host immune response to lytic viral infection [48]. In the lytic infection BZLF1 inhibits the activity of the promoter for the gene encoding TNF-α receptor and decreased expression of the TNFR1 protein [49]. Besides, it is unable to induce cellular apoptosis. TNF-α activates expression of various important inflammatory genes and it induces apoptosis.

BZLF1 stimulates expression of both TGF-β and IL-10 [49]. Epstein-Barr virus late gene BCRF1 is very similar to the human IL-10 gene (84 % homology in amino acids sequence). This late viral gene product vIl-10 (BCLF1) plays an important role for the protection of the virus from the host immune response. The results of this process, namely the infection with EBV, may develop tumour diseases.

Many questions are unclear and will need to be answered by future studies. All the above-mentioned cytokines and growth factors act on the tumour growth. Some of them were definitely studied and explained long ago, some are constantly being investigated. Explaining the reasons for this influence is very difficult because many cytokines interact in various ways on cells, often in an antagonistic manner, depending on the concentration. There is no doubt that viruses which have the ability to connect with the genetic material of the host cells can significantly disrupt gene expression for cytokines and growth factors.

It has been confirmed in numerous studies that HPV and EBV have the ability to cause various cancers, but there is a lack of studies which determine variability in the levels of cytokines in patients with OSCC with EBV infections.

## Conclusions

Our results clearly indicate increased levels of IL-10, TNF-α, TGF-β and VEGF both in serum and saliva and they suggest that these cytokines may constitute valuable tools in diagnosis, treatment and prognosis in cancer disease. However, further research is still required to prove the role and mode of action of potential biomarkers.

## Abbreviations

CSIF, cytokine synthesis inhibitor factor; DNA, deoxyribonucleic acid; EBNA-2, Epstein-Barr nuclear antigen 2; EBV, Epstein-Barr virus; ELISA, enzyme-linked immunosorbent assay; GCP, good clinical practice; HPV, human papillomavirus; IARC, International Agency for Research on Cancer; IL-10, interleukin 10; mRNA, messenger ribonucleic acid; OSCC, oral squamous cell carcinoma; PCR, polymerase chain reaction; SCC, squamous cell carcinoma; TGF-β, transforming growth factor beta; TNF-α, tumour necrosis factor alpha; TNM, tumour, node, metastasis; VEGF, vascular endothelial growth factor

## References

[1] Alibek K, Kakpenova A, Baiken Y (2013). Role of infectious agents in the carcinogenesis of brain and head and neck cancers. Infect Agent Cancer.

[2] Prasad G, McCullough M. Chemokines and cytokines as salivary biomarkers for the early diagnosis of oral cancer. Int J Dent. 2013, doi: 10.1155/2013/813756. 10.1155/2013/813756 PMC3860143 24376459

[3] Fakhry C, Westra WH, Li S, Cmelak A, Ridge JA, Pinto H (2008). Improved survival of patients with human papillomavirus-positive head and neck squamous cell carcinoma in a prospective clinical trials. J Natl Cancer Inst.

[4] Gillison ML, Koch WM, Capone RB, Spafford M, Westra WH, Wu L (2000). Evidence for a causal association between human papillomavirus and a subset of head and neck cancers. J Natl Cancer Inst.

[5] Hillbertz NS, Hirsch JM, Jalouli J, Jalouli M, Sand L (2012). Viral and molecular aspects of oral cancer. Anticancer Res.

[6] Scully C (2011). Oral cancer aetiopathogenesis; past, present and future aspects. Med Oral Patol Oral Cir Bucal.

[7] Alibek K, Baiken Y, Kakpenova A, Mussabekova A, Zhussupbekova S, Akan M (2014). Implication of human herpesviruses in oncogenesis through immune evasion and supression. Infect Agent Cancer.

[8] Schiller JT, Lowy DR (2014). Virus infection and human cancer: an overview. Recent Results Cancer Res.

[9] IARC monographs on the evaluation of carcinogenic risks to humans. World Health Organization. Lyon, France, 2007; 222–235.

[10] IARC monographs on the evaluation of carcinogenic risks to humans. A review of human carcinogens. Biological agents. World Health Organization. Lyon, France, 2012; 255–275.

[11] Acharya S, Ekalaksananan T, Vatanasapt P, Loyha K, Phusingha P, Promthet S (2015). Association of Epstein-Barr virus infection with oral squamous cell carcinoma in a case–control study. J Oral Pathol Med.

[12] Jalouli J, Ibrahim SO, Mehrotra R, Jalouli MM, Sapkota D, Larson PA, Hirsch JM (2010). Prevalence of viral (HPV, EBV, HSV) infections in oral submucous fibrosis and oral cancer from India. Acta Otolaryngol.

[13] Jalouli J, Jalouli MM, Sapkota D, Ibrahim SO, Larson PA, Sand L (2012). Human papilloma virus, herpes simplex virus and Epstein-Barr virus in oral squamous cell carcinoma from eight different countries. Anticancer Res.

[14] Kis A, Feher K, Gall T, Tar I, Boda R, Toth ED (2009). Epstein-Barr virus prevalence in oral squamous cell cancer and potentially malignant oral disorders in an eastern Hungarian population. Eur J Oral Sci.

[15] Mascolo M, Siano M, Ilardi G, Russo D, Merolla F, Rosa GD (2012). Epigenetic Disregulation in Oral Cancer. Int J Mol Sci.

[16] Jin L, Sturgis EM, Zhang Y, Huang Z, Song X, Chao X (2013). Association of tumor necrosis factor-alpha promoter variants with risk of HPV-associated oral squamous cell carcinoma. Mol Cancer.

[17] Elashoff D, Zhou H, Reiss J, Wong J, Xiao H, Henson B (2012). Prevalidation of salivary biomarkers for oral cancer detection. Cancer Epidemiol Biomarkers Prev.

[18] Korostoff A, Reder L, Masood R, Sinha UK (2011). The role of salivary cytokine biomarkers in tongue cancer invasion and mortality. Oral Oncol.

[19] Moser DM, Zhang X (2008). Interleukin-10: new perspectives on an old cytokine. Immunol Rev.

[20] Torres-Poveda K, Bahena-Román M, Madrid-González C, Burquete-Garcia AI, Bermudez-Morales VH, Peralta-Zaragoza O (2014). Role of IL-10 and TGF-β1 in local immunosuppression in HPV-associated cervical neoplasia. World J Clin Oncol.

[21] Jin L, Sturgis EM, Cao X, Song X, Salahuddin T, Wei Q (2013). Interleukin-10 promoter variants predict HPV-positive tumors and survival of squamous cell carcinoma of the oropharynx. FASEB J.

[22] Goncalves AS, Arantes DA, Bernardes VF, Jaeger F, Silvia JM, Silvia TA (2015). Immunosuppresive mediators of oral squamous cell carcinoma in tumour samples and saliva. Hum Immunol.

[23] Jiang C, Ye D, Qiu W, Zhang X, Zhang Z, He D (2007). Response of lymphocyte subsets and cytokines to Shenyang prescription in Sprague–Dawley rats with tongue squamous cell carcinomas induced by 4NQO. BMC Cancer.

[24] Lathers DM, Young MR (2004). Increased aberrance of cytokine expression in plasma of patients with more advanced squamous cell carcinoma of the head and neck. Cytokine.

[25] Wajant H, Pfizenmaier K, Scheurich P (2003). Tumor necrosis factor signaling. Cell Death Differ.

[26] Mocellin S, Nitti D (2008). TNF and cancer: the two sides of the coin. Front Biosci.

[27] Krishnan R, Thayalan DK, Padmanaban R, Ramadas R, Annasamy RK, Anandan N (2014). Association of serum and salivary tumor necrosis factor-α with histological grading in oral cancer and its role in differentiating premalignant and malignant oral disease. Asian Pac J Cancer Prev.

[28] Juretić M, Cerović R, Belusoć-Gobić M, Brekalo Prso I, Kqiku L, Spalj S (2013). Salivary levels of TNF-α and IL-6 in patients with oral premalignant and malignant lesions. Folia Biol.

[29] Rhodus NL, Ho V, Miller CS, Myers S, Ondrey F (2005). NF-kappaB dependent cytokine levels in saliva of patients with oral preneoplastic lesions and oral squamous cell carcinoma. Cancer Detect Prev.

[30] Piva MR, DE Souza LB, Martins-Filho PR, Nonaka CF, DE Santana ST, DE Souza Andrade ES (2013). Role of inflammation in oral carcinogenesis (Part II): CD8, FOXP3, TNF-α. TGF-β and NF-kB expression Oncol Lett.

[31] Massagué J (2008). TGFβ in Cancer. Cell.

[32] Mincione G, Di Marcantonio MC, Artese L, Vianale G, Piccirelli A, Piccirelli M (2008). Loss of expression of TGF-beta1, TbetaRI and TbetaRII correlates with differentiation in human oral squamous cell carcinomas. Int J Oncol.

[33] Friedrich RE, Klapdor R, Hagel C, Bartel-Friedrich S (2010). Vascular endothelial growth factor (VEGF) in sera of oral and oropharyngeal squamous cell carcinoma patients. Anticancer Res.

[34] Upile T, Jerjes W, Kafas P, Harini S, Singh SU, Guyer M (2009). Salivary VEGF: a non-invasive angiogenic and lymphangiogenic proxy in head and neck cancer prognostication. Int Arch Med.

[35] Ding L, Hu EL, Xu YJ, Huang XF, Zhang DY, Li B (2015). Serum IL-17F combined with VEGF as potential diagnostic biomarkers for oral squamous cell carcinoma. Tumour Biol.

[36] Aggarwal S, Devaraja K, Sharma SC, Das SN (2014). Expression of vascular endothelial growth factor (VEGF) in patients with oral squamous cell carcinoma and its clinical significance. Clin Chim Acta.

[37] Andisheh-Tadbir A, Hamzavi M, Rezvani G, Ashraf MJ, Fattahi MJ, Khademi B (2014). Tissue expression, serum and salivary levels of vascular endothelial growth factor in patients with HNSCC. Braz J Otorhinolaryngol.

[38] Czerninski R, Basile JR, Kartin-Gabay T, Laviv A, Barak V (2014). Cytokines and tumor markers in potentially malignant disorders and oral squamous cell carcinoma: a pilot study. Oral Dis.

[39] Tribius S, Hoffmann M (2013). Human papilloma virus infection in head and neck cancer. Dtsch Arztebl Int.

[40] Guan X, Sturgis EM, Lei D, Liu Z, Dahlstrom KR, Wei Q (2010). Association of TGF-beta1 genetic variants with HPV16-positive oropharyngeal cancer. Clin Cancer Res.

[41] Chuang CY, Sung WW, Wang L, Lin WL, Yeh KT, Su MC (2012). Differential impact of IL-10 expression on survival and relapse between HPV16-positive and -negative oral squamous cell carcinomas. PLoS One.

[42] Bermudez-Morales VH, Gutierrez LX, Alcocer-Gonzalez JM, Burguete A, Madrid-Marina V (2008). Correlation between IL-10 gene expression and HPV infection in cervical cancer: a mechanism for immune response escape. Cancer Invest.

[43] Jo S, Juhasz A, Zhang K, Ruel C, Loera S, Wilczynski SP (2009). Human papillomavirus infection as a prognostic factor in oropharyngeal squamous cell carcinomas treated in a prospective phase II clinical trial. Anticancer Res.

[44] Fei J, Hong A, Dobbins TA, Jones D, Lee CS, Loo C (2009). Prognostic significance of vascular endothelial growth factor in squamous cell carcinomas of the tonsil in relation to human papillomavirus status and epidermal growth factor receptor. Ann Surg Oncol.

[45] Chen CJ, Sung WW, Su TC, Chen MK, Wu PR, Yeh KT (2013). High expression of interleukin 10 might predict poor prognosis in early stage oral squamous cell carcinoma patients. Clin Chim Acta.

[46] Shi Y, Peng SL, Yang LF, Chen X, Tao YG, Cao Y (2016). Co-infection of Epstein-Barr virus and human papillomavirus in tumorigenesis. Chin J Cancer.

[47] Odumade OA, Hogquist KA, Balfour HH (2011). Progress and problems in understanding and managing primary Epstein-Barr virus infections. Clin Microbiol Rev.

[48] Che AV, Lopez P, Pandofli PP, Roizman B (2003). Promyelocytic leukemia protein mediates interferon-based anti-herpes simplex virus 1 effects. J Virol.

[49] Morrison TE, Mauser A, Klingelhutz A, Kenney SC (2004). Epstein-Barr virus immediate-early protein BZLF1 receptor. J Virol.

